# COVID-19 Vaccination Status and Hesitancy among Breast Cancer Patients after Two Years of Pandemic: A Cross-Sectional Survey

**DOI:** 10.3390/vaccines10091530

**Published:** 2022-09-15

**Authors:** Weijing Liu, Yunhao Wu, Ruoning Yang, Ruixian Chen, Ya Huang, Xin Zhao, Min Xie, Qintong Li, Qiang Wang, Jie Chen

**Affiliations:** 1 Department of Breast Center, West China Hospital, Sichuan University, Chengdu 610041, China; 2 Mental Health Centre, West China Hospital, Sichuan University, Chengdu 610041, China; 3 Departments of Obstetrics & Gynecology and Pediatrics, West China Second University Hospital, Key Laboratory of Birth Defects and Related Diseases of Women and Children, Ministry of Education, Development and Related Diseases of Women and Children Key Laboratory of Sichuan Province, Sichuan University, Chengdu 610041, China

**Keywords:** COVID-19, booster vaccination, adverse event, breast cancer, vaccine hesitancy

## Abstract

**Simple Summary:**

The coronavirus disease 2019 (COVID-19) vaccine has played an important role in preventing infection and adverse outcomes in immunocompromised cancer patients, especially in breast cancer, one of the common tumor types. However, there are limited reports of vaccination in breast cancer patients and these are limited by the timeliness of research investigations. This is a cross-sectional study of 1132 Chinese breast cancer patients aged 18 years and older, each of whom completed an online self-reported questionnaire. This study was conducted to describe the vaccination status, adverse effects and vaccine hesitancy towards future vaccinations or boosters among Chinese breast cancer patients after two years of the pandemic, to provide new perspectives for health promotion.

**Abstract:**

Background: Patients with cancer show greater susceptibility and vulnerability to severe acute respiratory syndrome coronavirus 2 infection. However, data on the vaccination status among patients with breast cancer and any structured analysis of the factors influencing patients’ decisions regarding vaccines are lacking. Methods: This cross-sectional study on patients with breast cancer in China was conducted from 1 June 2022, to 17 June 2022. Every participant completed an online questionnaire about their vaccination status and any adverse reactions, and a scale based on the Health Belief Model (HBM) to assess the vaccination status of respondents and their willingness to receive following doses or boosters. Results: Among the 1132 participants, 55.2% had received a COVID-19 vaccine. The incidence of adverse events per dose was around 40%. Vaccine hesitancy of 61.9% was observed among patients who had not fully received three doses of vaccine or boosters. The only variable found to be associated with vaccine hesitancy was time since diagnosis (*p* < 0.05). In the HBM scale, vaccine hesitancy was closely related to a low level of perceived susceptibility, a low level of perceived benefit, a high level of perceived barriers and a low level of agreement with doctors’ advice. Conclusions: For patients with breast cancer, perceived susceptibility, benefits and barriers should be prioritized, and the advice from authoritative doctors is a vital cue to action.

## 1. Introduction

With the global epidemic of coronavirus disease 2019 (COVID-19) continuing for more than 2 years, patients with cancer show increased susceptibility and vulnerability due to their immunocompromised condition and have a high risk of developing COVID-19 related complications [1,2,3,4]. High mortality has been recorded among patients with solid or hematological malignancies infected with severe acute respiratory syndrome coronavirus 2 (SARS-CoV-2) [5,6]. As one of the largest cancer populations, patients with breast cancer account for a high percentage of SARS-CoV-2 infections [7,8] and thus require attention and medical support during the COVID-19 pandemic.

Patients with cancer should be considered a high-risk group for priority COVID-19 vaccination [9]. Despite the considerable uncertainty in vaccine efficacy, immune responses and adverse events, the UK Coronavirus Cancer Evaluation Project and other studies have supported the recommendation that a third dose or booster should be prioritized in patients with cancer because vaccine effectiveness declines faster in these groups than in the general population [10,11].

Vaccination rates among cancer patients remain low [6,12], which is indicative of hesitation regarding the vaccine [13,14]. Vaccine hesitancy refers to the delay in acceptance or refusal of safe vaccines despite the availability of vaccination services, and this phenomenon poses a major threat to global health [15]. Studies on vaccination status and attitudes toward COVID-19 vaccines among the cancer population in various countries have reported the incidences of vaccine hesitancy to be around 18–74% [16,17,18,19,20]. As the COVID-19 pandemic continues to ravage communities worldwide, the threat of vaccine hesitancy has become evident and urgent.

Current studies on the vaccination willingness of patients with breast cancer provide limited data on patient vaccination rates and self-reported adverse events. Any structured analysis of the reasons influencing patients’ decision regarding vaccines is also lacking. Thus, we conducted a cross-sectional survey of patients with breast cancer to investigate the current vaccination status and reported adverse events among the vaccinated population. Considering that the vaccination recommendation for COVID-19 worldwide is full vaccination with a booster dose, we designed a Health Belief Model (HBM)-based scale to analyze the cognitive and behavioral patterns in response to future vaccinations among individuals who have not received full vaccination or boosters. The HBM is a common tool to predict intention and behavior for influenza vaccination [21,22,23] and contains different dimensions, such as perceived susceptibility, perceived severity, perceived benefits, perceived barriers and cues to action. Vaccine hesitation has been associated with HBM dimensions [24,25].

With this study, we hope to provide evidence-based data to address the concerns of patients with breast cancer and to improve the coverage of the COVID-19 vaccination.

## 2. Methods

### 2.1. Study Design and Participants

This internet-based, cross-sectional study was conducted among patients with breast cancer in West China Hospital. The questionnaire was administered to individuals and collected via an online platform from 1 June 2022, to 17 June 2022. The study purpose was explained to these individuals and informed consent was obtained before the survey. The inclusion criteria for patients were as follows: (1) >18 years old, (2) diagnosed with breast cancer via a pathology test and (3) agreed to participate. This study was approved by the Clinical Test and Biomedical Ethics Committee of West China Hospital, Sichuan University (approval number: 2022649).

### 2.2. Survey and Data Collection

The questionnaire was designed by a panel of psychologists and clinicians and consisted of three parts: (1) demographics and clinical history, (2) vaccination status and adverse event and (3) vaccine hesitancy and HBM scale. The HBM scale was adopted from a similar work on vaccine acceptance and was proven to be effective and feasible [26]. Patients who had not been vaccinated yet or who had received one or two doses were instructed to answer relevant questions on their willingness to receive a future COVID-19 dose/booster in the scale, followed by a 5-point Likert HBM scale (1-strongly disagree, 5-strongly agree).

**Demographics and clinical history**: Sociodemographic information included age, marital status, education, number of family members, annual income (USD) and medical costs. Clinical history included time since diagnosis (year), comorbidity, number of comorbidities, treatment type and number of treatments.

**Vaccination status and adverse event**: Data on vaccination status, doses, types, time (before or after diagnosis) and adverse events for each dose were collected through several questions. Patients who received at least one dose of vaccine were asked to report any immediate (<48 h) and late (>48 h) adverse events.

**Vaccine hesitancy and HBM scale**: Patients who had not received the third dose/booster were asked about their willingness to receive future vaccination (Table S1). Cronbach’s alpha was calculated for the questions of each construct of the HBM to determine the internal consistency and reliability of our questionnaire. The HBM scale consisted of 18 items of 5 dimensions: perceived susceptibility (1–3 items; Cronbach’s alpha, 0.735), perceived severity (4–7 items; Cronbach’s alpha, 0.818), perceived benefits (8–10 items; Cronbach’s alpha, 0.856), perceived barriers (11–15 items; Cronbach’s alpha, 0.874) and cues to action (16–18 items; Cronbach’s alpha, 0.782). In general, a Cronbach’s alpha of more than 0.7 indicates the internal consistency of the item groups. A Kaiser–Meyer–Olkin test (0.757) and Bartlett’s test of sphericity (*p* < 0.001) confirmed the adequacy of the sample for exploratory factor analysis.

### 2.3. Statistical Analysis

Data analysis was performed using IBM SPSS statistics software (IBM Corp. Hp Chicago, IL, USA. Version 24.0) and Tableau (Tableau Software, Seattle, WA, USA. Version 2021.1). Depending on the type of the variable, a chi-square test, Fisher’s exact test or Student’s *t*-test was used to compare the distribution of baseline characteristics among all patients. The dependent variable “Are you willing to get the future vaccination?” had two possible values: 0 = (“willing to be vaccinated”); 1 = (“hesitate/refuse to be vaccinated”). The scores for the HBM scale in each item were transformed to binary variables for logistic regression: 1 = (“strongly agree/agree”); 0 = (“neutral/disagree/strongly disagree”). Forward univariate and multivariate analyses based on Pearson’s chi-square tests were performed to determine the association between sociodemographic and clinical variables and reported adverse events/vaccine acceptance, and the values were presented as odds ratios (OR) with a 95% confidence interval (95% CI). All statistical tests were 2-tailed and *p* values < 0.05 were considered to be statistically significant.

## 3. Results

### 3.1. Sociodemographic and Clinical Characteristics of Participants

A total of 1170 questionnaires were collected, of which 38 duplicated questionnaires were excluded. Finally, 1132 patients were included in this study.

Among all the patients, 625 (55.2%) received at least one dose of the COVID-19 vaccine and 507 (44.8%) had not been vaccinated. Among the vaccinated patients, 3.36% (21/625) received only one dose, 51.36% (321/625) received two doses and 45.28% (283/625) completed the third dose. A total of 453 patients reported their vaccine types, among which 436 (96.2%) received inactivated vaccines (including Beijing Institute of Biological Products Co., Ltd. Beijing, China, Wuhan Institute of Biological Products Co., Wuhan, China and Sinovac Life Sciences Co., Ltd. Beijing, China), 4 (0.9%) received adenovirus vaccines (CanSino Biologics Inc. Tianjin, China) and 13 (2.9%) received recombinant vaccines (Anhui Zhifei Longcom Biopharmaceutical Co., Ltd. Hefei, China). Relevant information regarding all reported vaccines is listed in Table S2.

The patients were divided into vaccinated and nonvaccinated groups according to their vaccination status (Table 1). The age of all patients ranged 25–84 years with a median age of 49.2 years. No significant difference in age composition was observed between the vaccinated and nonvaccinated groups (*p* = 0.183), and 74.4% of all patients were between 40 and 59 years of age. The majority of patients who participated in the questionnaire (71.8%) were diagnosed <3 years ago, and nearly half of the vaccinated patients had been diagnosed within 1 year (56.0%). Among all the participants, 91.1% were married; 70.3% had high-school education or above; 87.1% had families with three or more members; 68.1% had an annual income of less than 15,000 USD; and 88.5% had medical insurance. These factors showed no statistically significant differences between the two groups.

Data on comorbidities and treatment modalities were also recorded. Nearly half of all the patients had at least one comorbidity. The vaccinated patients seemed to have fewer comorbidities than the nonvaccinated patients (62.2% vs. 49.7%, *p* < 0.001). Among the patients with comorbidities, hyperlipidemia (11.4%) and hypertension (10.2%) were the most common diseases, followed by diabetes/dysglycemia (6.9%), rheumatism and asthma (6.7%) and gastrointestinal disorders (6.3%). Patients who had been receiving at least two treatment modalities accounted for 48.8%. Endocrine therapy (56.0%) and surgery (51.1%) had the largest proportion and accounted for more than half of all the patients, followed by chemotherapy (38.3%), targeted therapy (17.8%) and radiotherapy (15.6%). Few patients (9.3%) had received no treatment at the time of the survey.

### 3.2. Self-Reported Adverse Events

**Figure 1:**
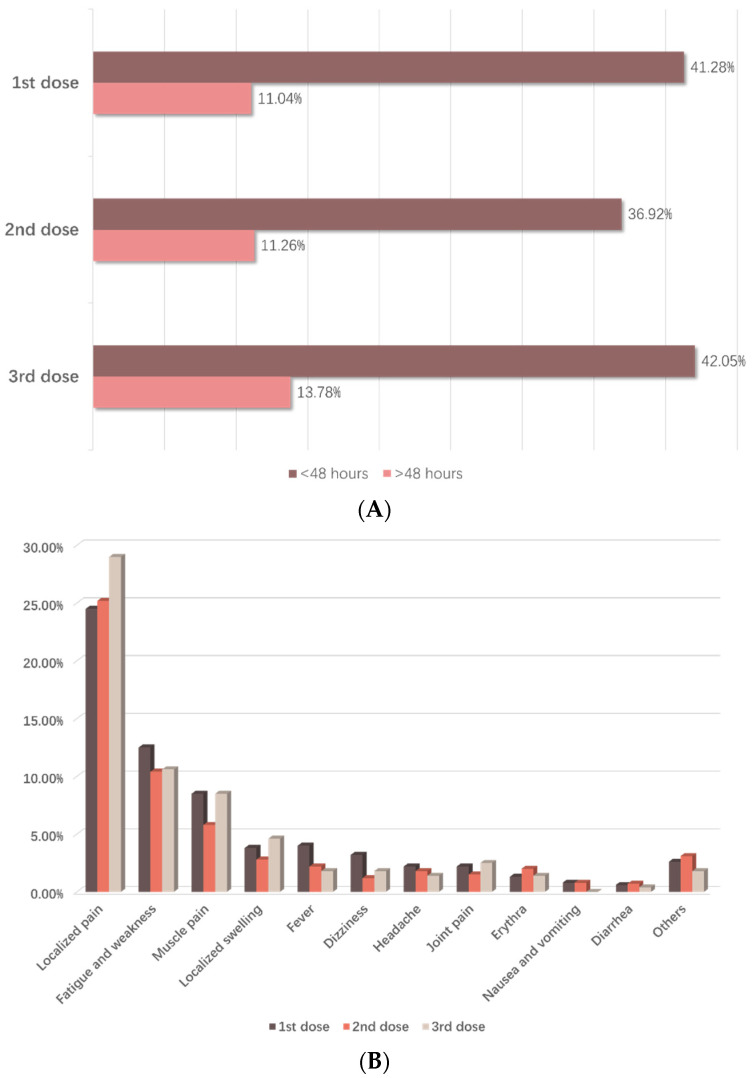
Self-reported Adverse Events among Vaccinated Patients. (**A**) Incidence of adverse events within and after 48 h for each dose. (**B**) Incidence of specific adverse events within 48 h for each dose in reported cases.

Among the 625 vaccinated patients, the incidence of adverse events was 41.60% (260/625), 38.08% (230/604) and 42.78% (121/283) for the first, second and third doses, respectively. The rate of vaccination adverse events after 48 h decreased significantly compared with the reported rate within 48 h (Figure 1A). Local pain had the highest proportion, followed by fatigue and muscle pain for the first, second and third doses. Other reported adverse events included localized swelling, fever and dizziness. The incidence of diarrhea, nausea and vomiting had the lowest proportion, at less than 1% (Figure 1B).

The association of individual and disease-related factors with adverse events after vaccination was analyzed using univariate and multivariate logistic regression. Table 2 shows that age, time since diagnosis, education, annual income, comorbidities and treatment modalities were all associated with adverse events in the multivariate analysis (Table 2). Old age was associated with decreased incidence of adverse events compared with <40 years of age (40–59 years of age: aOR, 0.292; 95% CI, 0.125–0.684; *p* = 0.005; >60 years of age: aOR, 0.107; 95% CI, 0.038–0.303, *p* < 0.001). Similarly, a time since diagnosis >4 years seemed to be a decreased risk factor for adverse events (time since diagnosis of 4–5 years: aOR.0.453, 95% CI, 0.218–0.941; *p* = 0.034; >5 years: aOR, 0.348; 95% CI, 0.177–0.682; *p* = 0.002). Comorbidity (one comorbidity: aOR, 3.851; 95% CI, 2.154–6.882; *p* < 0.001; two or more comorbidities: OR, 3.099; 95% CI, 1.391–6.902; *p* = 0.006), receiving endocrine therapy (aOR, 2.157; 95% CI, 1.125–4.136; *p* = 0.021) and targeted therapy (aOR, 2.474; 95% CI, 1.134–5.397; *p* = 0.023) were all associated with an increased risk of adverse events. High educational attainment seemed to be associated with an increased incidence of self-reported adverse events (bachelor’s degree and above: aOR, 2.669; 95% CI, 1.200–5.938; *p* = 0.016).

### 3.3. Willingness to Receive Future Vaccination

**Figure 2:**
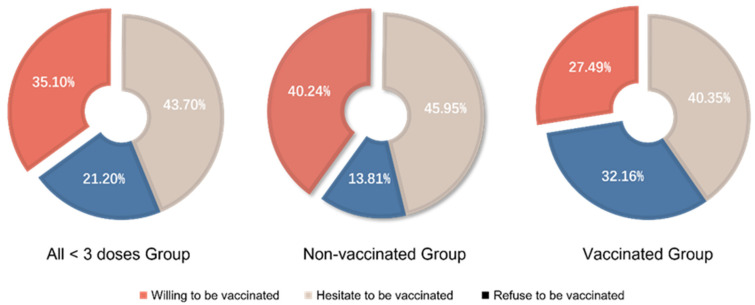
Willingness regarding future doses/boosters in all <3 doses group, non-vaccinated group and vaccinated group.

Willingness to receive a future vaccination/booster shot was studied among the 849 patients who had not complete all three doses of vaccination. Among them, 298 (35.10%) patients were willing to receive an injection in the future. The nonvaccinated patients showed higher acceptance than the vaccinated group (40.24% vs. 27.49%, *p* < 0.001) (Figure 2).

Univariate and multivariate logistic regression analyses were performed to analyze the factors influencing patients’ willingness to receive future vaccination (Table 3). Only time since diagnosis was found to be associated with hesitancy after multivariate analysis. The risk of vaccine hesitancy decreased with the increasing time since diagnosis compared with that within 1 year (time since diagnosis of 1–3 years: aOR, 0.192; 95% CI, 0.115–0.321; *p* < 0.001; 4–5 years: aOR, 0.479; 95% CI, 0.260–0.884; *p* = 0.019; >5 years: aOR, 0.408; 95% CI, 0.215–0.775; *p* = 0.006).

**Figure 3:**
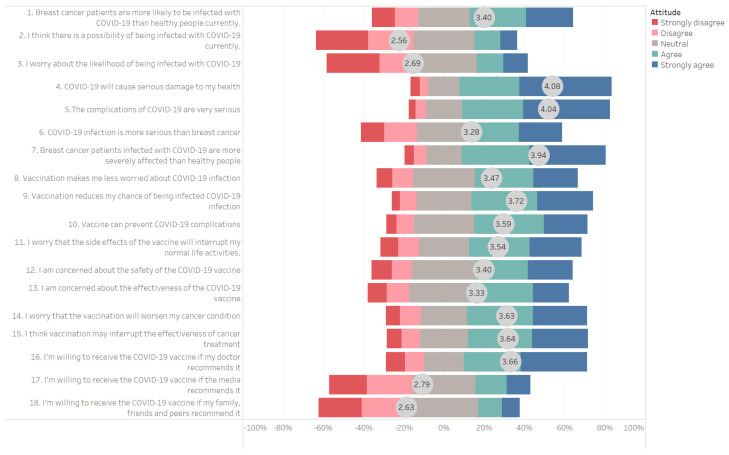
Patients perceived vaccine hesitancy towards the COVID-19 vaccine (Based on Healthy Belief Model).

An HBM-based 5-point Likert scale was used to investigate the perceptions among these patients. The distribution and the mean score for each question are shown in Figure 3. A score > 3 means that the patient agrees with the view, and a score < 3 means that the patient disagrees with the view. In this study, question 4 “COVID-19 will cause serious damage to my health,” question 5 “The complications of COVID-19 are very serious” and question 7 “Patients with breast cancer infected with COVID-19 are more severely affected than the rest of the population” had a mean score > 3 (neutral). Question 2 “I think there is a possibility of being infected with COVID-19 currently,” question 3 “I worry about the likelihood of being infected with COVID-19,” question 17 “I’m willing to receive the COVID-19 vaccine if the media recommends it” and question 18 “I’m willing to receive the COVID-19 vaccine if my family, friends and peers recommend it” had a mean score < 3 (neutral).

**Figure 4:**
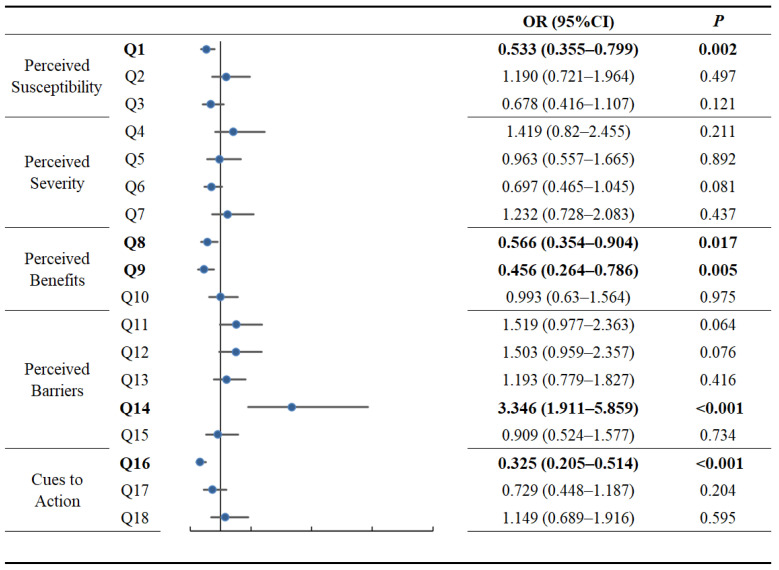
Adjusted Odds Ratio (OR) of comparing the rate of COVID-19 vaccine hesitancy within participants with different items. Statistically significant results (*p* < 0.05) are in bold font.

Figure 4 shows the multivariate analysis result of patients’ perceptions and hesitancy (Figure 4). The following four questions were significantly associated with decreased hesitancy: Q1 “I believe that patients with breast cancer are more likely to be infected with COVID-19 than healthy people currently” (perceived susceptibility) (aOR, 0.533; 95% CI, 0.355–0.799, *p* = 0.002); Q8 “Vaccination makes me less worried about COVID-19 infection” (perceived benefits) (aOR, 0.566; 95% CI, 0.354–0.904, *p* = 0.017); Q9 “Vaccination reduces my chance of being infected COVID-19 infection” (perceived benefits) (OR, 0.456; 95% CI, 0.264–0.786, *p* = 0.005); Q16 “I’m willing to receive the COVID-19 vaccine if my doctor recommends it” (action cues) (aOR, 0.325; 95% CI, 0.205–0.514, *p* < 0.001). Fear of the vaccine’s impact on their cancer condition (Q14, perceived barriers; aOR, 3.346; 95% CI, 1.911–5.859, *p* < 0.001) was a significant risk factor for hesitancy and refusal of future vaccination.

## 4. Discussion

The COVID-19 vaccine has played an important role in the prevention and control of the epidemic in the past 2 years. Especially for patients with cancer, vaccination has reduced their adverse outcomes (morbidity, mortality, sequelae, ICU admission and COVID-19 severity) [27,28]. However, vaccine hesitancy remains high in oncology patients, leading to low vaccination rates and an increased medical burden. This study investigated the vaccination status of patients with breast cancer and their cognitive and behavioral patterns regarding vaccine hesitancy.

China was one of the first countries to report COVID-19 cases and successfully develop vaccines. About 3.4 billion vaccine doses have been administered up to 30 June 2022, suggesting a more than 90% vaccination rate among all populations. In our study, only 55.2% of patients were vaccinated. This rate was lower than the current vaccination rate in the general population. Nearly half of the vaccinated patients were within their 1st year of diagnosis, possibly because they were diagnosed with breast cancer only after being vaccinated.

The adverse event rate for each dose was 40% in our study population. Tsai et al. [8] reported a 69.0% rate of local reactions and a 40.0% rate of systemic reactions among individuals with cancer and autoimmune diseases [18]. In a prospective study in Germany, the incidence of post-vaccination adverse events was 63.3% (first dose) and 71.2% (second dose) [29]. The higher incidence of these adverse reactions compared with that in our study may be related to the use of different brands of vaccine. For example, the Pfizer-BioNTech vaccine is the most commonly used brand worldwide but is underutilized in China.

Our results showed that the most common adverse effects after injection were local pain, fatigue and weakness and muscle pain. The incidence of these events was significantly lower 48 h after vaccination, and this result is in agreement with other studies. A patient age > 40 years was associated with reduced adverse events compared with younger age groups. This finding is consistent with that of another study, which indicated that adverse reactions were more common in younger age groups than in older age groups [30]. This difference may be explained by the reduced immune responses in the older patients due to immunosenescence [31]. In addition, a short time since cancer diagnosis and the presence of two or more comorbidities, as well as receipt of endocrine therapy or targeted therapy, were all associated with an increased incidence of adverse events. However, no serious adverse reactions were reported by the patients.

A vaccine hesitancy rate of approximately 61.9% was reported among the patients who had not yet completed the vaccination/booster; this value is higher than that in other studies (18–74%) [16,17,18,19,20]. Our study showed that a shorter time since diagnosis is a risk factor for vaccine hesitancy. Previous reports have included additional risk factors [17,32], such as a prior history of COVID-19 infection (OR 0.86), a younger age (OR 0.83), conservative political leanings (OR 0.93) and a low education level (OR 0.90).

Logistic regression analysis suggested that perceived susceptibility (Q1, *p* = 0.002), perceived benefit (Q8, *p* = 0.017; Q9, *p* = 0.005), perceived barriers (Q14, *p* < 0.001) and cues to action (Q16, *p* < 0.001) were significantly associated with vaccine hesitancy. Consistent with our findings, Lai et al. [33] reported that the perceived benefits and barriers are important dimensions associated with willingness to receive COVID-19 booster shots. Regarding perceived susceptibility, participants’ perceptions of the risk of SARS-CoV-2 infection tended to be neutral or disagreeable, possibly due to the effective control of the pandemic in China. This intriguing finding may be related to the fallback to normalcy of people’s negative emotions as the epidemic progresses [34]. However, our study found that the individuals’ perceptions of their own risk of infection as patients with cancer were associated with decreased hesitancy. In terms of perceived barriers, patients’ fearful perceptions of possible adverse effects on their cancer were associated with increased hesitancy, and this finding is consistent with results from Turkey [35]. Compared with the media, family, friends and peers, doctor’s advice was significantly associated with decreased vaccine hesitancy. Consistent with other studies, this result suggests that authoritative advice from doctors is an important contributor to patients’ willingness to be vaccinated [17]. Patients have different considerations about their diseases than those of the general population when it comes to vaccination. The core cognitive patterns of patients’ attitudes towards vaccination included the following: belief that they were at higher risk of infection compared to healthy people; a lack of perceived benefit from the vaccination; and fear of any possible negative impacts of the vaccine on their health during their disease. These findings indicate that the information regarding COVID-19 vaccine that is disseminated about among breast cancer patients should be different from that which is targeted at other populations, and that perceived susceptibility, benefits and barriers should be emphasized. In addition, advice from authoritative doctors is a vital cue to action.

### Strengths and Limitations

This work is the first cross-sectional study of COVID-19 vaccination among patients with breast cancer to capture and analyze such a large volume of data. This research also furthers efforts to increase the vaccination rate among high-risk populations according to the recommendations of the European Society of Medical Oncology [36], the American Society of Clinical Oncology [37] and the National Comprehensive Cancer Network [38]. In addition, details of self-reported adverse events were disclosed and analyzed. Finally, the HBM scale was used to investigate patients’ intentions regarding future vaccinations, providing evidence and recommendations for the current vaccine rollout and behavioral intervention guidance.

This study has some limitations. First, due to the single-centered online nature of the survey, selection bias may affect the generalizability of the results, especially given the participation of relatively few respondents with low educational attainment and fewer older adults (>70 years). Second, this cross-sectional study may not reflect future trends. Third, the multivariables of willingness and attitudes, comprising the 18 items of the scale, were compressed into binary variables (willingness vs. hesitation/refusal, agreement vs. neutrality/disagreement), possibly causing some information loss.

## 5. Conclusions

In conclusion, our results show that 55.2% of patients with breast cancer received a COVID-19 vaccine. The incidence of adverse events per dose was around 40%. The rate of vaccine hesitancy was 61.9% among the patients who had not received full vaccination or boosters. For patients with breast cancer, their perceived susceptibility and perceived barriers should be prioritized, and advice from authoritative doctors is a vital cue to action.

## Figures and Tables

**Table 1:** Baseline characteristics of patients.

Variables	Vaccinated *n* = 625 (%)	Non-Vaccinated *n* = 507 (%)	Total *n* = 1132 (%)	*p*-Value
Age, year				0.183
<40	105 (16.8)	67 (13.2)	172 (15.2)	
40–59	460 (73.6)	382 (75.3)	842 (74.4)	
≥60	60 (9.6)	58 (11.4)	118 (10.4)	
Time since diagnosis, year				<0.001
<1	350 (56.0)	30 (5.9)	380 (33.6)	
1–3	84 (13.4)	348 (68.6)	432 (38.2)	
4–5	82 (13.1)	80 (15.8)	162 (14.3)	
>5	109 (17.4)	49 (9.7)	158 (14.0)	
Marital status				0.335
Married	576 (92.2)	455 (89.7)	1031 (91.1)	
Unmarried	16 (2.6)	15 (3.0)	31 (2.7)	
Others	33 (5.3)	37 (7.3)	70 (6.2)	
Education				0.594
Middle school and below	197 (31.5)	139 (27.4)	336 (29.7)	
High school	101 (16.2)	89 (17.6)	190 (16.8)	
Technical secondary school	45 (7.2)	44 (8.7)	89 (7.9)	
College	148 (23.7)	123 (24.3)	271 (23.9)	
Bachelor and above	134 (21.4)	112 (22.1)	246 (21.7)	
Members in family				0.068
1–2	74 (11.8)	72 (14.2)	146 (12.9)	
3	259 (41.4)	230 (45.4)	489 (43.2)	
4	149 (23.8)	90 (17.8)	239 (21.1)	
≥5	143 (22.9)	115 (22.7)	258 (22.8)	
Annual income, USD				0.382
<3000	149 (23.8)	121 (23.8)	270 (23.9)	
3000–15,000	278 (44.5)	223 (44.0)	501 (44.3)	
15,000–30,000	125 (20.0)	117 (23.1)	242 (21.4)	
>30,000	73 (11.7)	46 (9.1)	119 (10.5)	
Medical cost				0.106
Self-paid	47 (7.5)	23 (4.5)	70 (6.2)	
Insurance	547 (87.5)	455 (89.7)	1002 (88.5)	
Others	31 (5.0)	29 (5.7)	60 (5.3)	
(Comorbidity)				
Hypertension	58 (9.3)	58 (11.4)	116 (10.2)	0.233
Dyslipidemia	65 (10.4)	64 (12.6)	129 (11.4)	0.242
Diabetes/Abnormal blood sugar	37 (5.9)	41 (8.1)	78 (6.9)	0.152
Chronic lung disease	19 (3.0)	32 (6.3)	51 (4.5)	0.008
Liver disease	21 (3.4)	26 (5.1)	47 (4.2)	0.138
Heart disease	11 (1.8)	12 (2.4)	23 (2.0)	0.472
Gastrointestinal disease	30 (4.8)	41 (8.1)	71 (6.3)	0.023
Kidney disease	13 (2.1)	15 (3.0)	28 (2.5)	0.344
Neuropsychiatric disease	15 (2.4)	18 (3.6)	33 (2.9)	0.253
Rheumatic disease and asthma	34 (5.4)	42 (8.3)	76 (6.7)	0.057
Number of comorbidities				<0.001
0	389 (62.2)	252 (49.7)	641 (56.6)	
1	173 (27.7)	173 (34.1)	346 (30.6)	
≥2	63 (10.1)	82 (16.2)	145 (12.8)	
(Treatment)				
Surgery	339 (45.8)	214 (57.8)	553 (51.1)	<0.001
Endocrine therapy	287 (45.9)	347 (68.4)	634 (56.0)	<0.001
Chemotherapy	314 (50.2)	119 (23.5)	433 (38.3)	<0.001
Radiotherapy	95 (15.2)	82 (16.2)	177 (15.6)	0.654
Targeted therapy	110 (17.6)	92 (18.1)	202 (17.8)	0.811
Others	22 (3.5)	36 (7.1)	58 (5.1)	0.007
Number of treatments				0.003
0	58 (9.3)	51 (10.1)	109 (9.6)	
1	234 (37.4)	237 (46.7)	471 (41.6)	
≥2	333 (53.3)	219 (43.2)	552 (48.8)	

**Table 2:** Univariable and Multivariable logistic regression for adverse events.

Characteristics	Univariable		Multivariable	
	OR (95% CI)	*p*	OR (95% CI)	*p*
Age, years				
<40	1			
40–59	0.264 (0.119–0.586)	<0.001	0.292 (0.125–0.684)	0.005
≥60	0.143 (0.056–0.364)	<0.001	0.107 (0.038–0.303)	<0.001
Time since diagnosis, year				
<1	1			
1–3	1.167 (0.595–2.290)	0.653	1.096 (0.499–2.406)	0.820
4–5	0.531 (0.302–0.933)	0.028	0.453 (0.218–0.941)	0.034
>5	0.429 (0.262–0.704)	0.001	0.348 (0.177–0.682)	0.002
Education				
Middle school and below	1			
High school	1.123 (0.637–1.978)	0.688	1.290 (0.676–2.461)	0.440
Technical secondary school	1.159 (0.535–2.511)	0.709	1.529 (0.651–3.589)	0.330
College	1.419 (0.841–2.394)	0.190	1.698 (0.886–3.256)	0.111
Bachelor and above	2.627 (1.404–4.915)	0.003	2.669 (1.200–5.938)	0.016
Annual income, yuan				
<20,000	1			
20,000–100,000	1.103 (0.680–1.790)	0.691	0.938 (0.533–1.649)	0.824
110,000–200,000	1.083 (0.680–1.790)	0.787	0.691 (0.331–1.442)	0.325
>200,000	2.023 (0.911–4.492)	0.083	0.980 (0.359–2.677)	0.969
Number of comorbidities				
0	1			
1	2.472 (1.469–4.159)	0.001	3.851 (2.154–6.882)	<0.001
≥2	1.713 (0.838–3.498)	0.140	3.099 (1.391–6.902)	0.006
Number of treatments				
0	1			
1	1.202 (0.636–2.274)	0.571	0.613 (0.266–1.412)	0.250
≥2	2.462 (1.295–4.682)	0.006	0.583 (0.178–1.915)	0.374
Surgery	1.971 (1.323–2.936)	0.001	1.692 (0.824–3.473)	0.152
Endocrine therapy	1.245 (0.837–1.851)	0.279	2.157 (1.125–4.136)	0.021
Chemotherapy	1.368 (0.922–2.029)	0.120	1.048 (0.547–2.009)	0.888
Radiotherapy	0.478 (0.247–0.926)	0.029	1.504 (0.703–3.216)	0.292
Targeted therapy	2.559 (1.327–4.934)	0.005	2.474 (1.134–5.397)	0.023

OR, odds ratio; CI, confidence interval.

**Table 3:** Univariable and Multivariable logistic regression for vaccine hesitancy.

	Univariable		Multivariable	
	OR (95% CI)	*p*	OR (95% CI)	*p*
Vaccination status				
Vaccinated	1		1	
Non-vaccinated	0.563 (0.419–0.757)	<0.001	1.306 (0.883–1.932)	0.181
Age, year				
<40	1			
40–59	0.920 (0.625–1.354)	0.672	1.117 (0.735–1.698)	0.604
≥60	0.848 (0.487–1.477)	0.561	0.987 (0.535–1.822)	0.966
Time since diagnosis, year				
<1	1			
1–3	0.239 (0.164–0.349)	<0.001	0.192 (0.115–0.321)	0.000
4–5	0.573 (0.340–0.967)	0.037	0.479 (0.260–0.884)	0.019
>5	0.495 (0.279–0.879)	0.016	0.408 (0.215–0.775)	0.006
Marital status				
Married	1			
Unmarried	1.686 (0.661–4.296)	0.274		
Others	1.498 (0.813–2.761)	0.195		
Education				
Middle school and below	1			
High school	0.920 (0.600–1.412)	0.704		
Technical secondary school	1.097 (0.609–1.940)	0.777		
College	1.137 (0.768–1.683)	0.522		
Bachelor and above	1.023 (0.687–1.522)	0.912		
Members in family				
1–2	1			
3	0.765 (0.488–1.199)	0.242		
4	0.799 (0.482–1.326)	0.386		
≥5	0.763 (0.465–1.252)	0.284		
Annual income, yuan				
<20,000	1			
20,000–100,000	0.883 (0.615–1.268)	0.501		
110,000–200,000	1.047 (0.685–1.599)	0.833		
>200,000	1.231 (0.718–2.111)	0.450		
Number of comorbidities				
0	1			
1	0.804 (0.589–1.098)	0.171	0.880 (0.631–1.225)	0.448
≥2	0.835 (0.548–1.273)	0.402	0.905 (0.568–1.440)	0.672
Number of treatments				
0	1			
1	0.593 (0.240–1.034)	0.065	0.567 (0.298–1.090)	0.084
≥2	0.700 (0.404–1.212)	0.203	0.480 (0.209–1.106)	0.085
Treatment				
Surgery	1.214 (0.915–1.611)	0.178	1.184 (0.684–2.048)	0.546
Endocrine therapy	0.639 (0.476–0.859)	0.003	1.035 (0.695–1.542)	0.864
Chemotherapy	1.459 (1.077–1.977)	0.015	1.195 (0.756–1.888)	0.445
Radiotherapy	0.932 (0.646–1.344)	0.707	0.827 (0.510–1.340)	0.440
Targeted therapy	0.958 (0.675–1.361)	0.811	0.823 (0.541–1.251)	0.362

OR, odds ratio; CI, confidence interval.

## Data Availability

The data that support the findings of this study are available on request from the corresponding author. The data are not publicly available due to privacy or ethical restrictions.

## Supplementary Materials

The following supporting information can be downloaded at: https://www.mdpi.com/article/10.3390/vaccines10091530/s1, Table S1: Scale of vaccine hesitancy; Table S2: Status of COVID-19 Vaccines within WHO EUL/PQ evaluation process (update on 7 July 2022).
