# Supplementary material for: COVID-19 Vaccination Status and Hesitancy among Breast Cancer Patients after Two Years of Pandemic: A Cross-Sectional Survey
Source: Vaccines (Basel). 2022 Sep 15;10(9):1530. doi: 10.3390/vaccines10091530 (PMC9503096; doi:10.3390/vaccines10091530)
Supplement: Supplementary file 1 [file vaccines-10-01530-s001.zip › vaccines-1852249-supplementary.pdf]

Table S1: Scale of vaccine hesitancy.

Score		1	2	3	4	5
		**Strongly disagree**	**Disagree**	**Neutral**	**Agree**	**Strongly agree**
**Perceived susceptibility**	1. I believe that breast cancer patients are more likely to be infected with COVID-19 than healthy people currently.					
	2. I think there is a possibility of being infected with COVID-19 currently.					
	3. I worry about the likelihood of being infected with COVID-19					
**Perceived severity**	4. COVID-19 will cause serious damage to my health					
	5. The complications of COVID-19 are very serious					
	6. COVID-19 infection is more serious than breast cancer					
	7. Breast cancer patients infected with COVID-19 are more severely affected than the rest of the population					
**Perceived benefits**	8. Vaccination makes me less worried about COVID-19 infection					
	9. Vaccination reduces my chance of being infected COVID-19 infection					
	10. Vaccine can prevent COVID-19 complications					
**Perceived barrier**	11. I worry that the side effects of the vaccine will interrupt my normal life activities.					
	12. I am concerned about the safety of the COVID-19 vaccine					
	13. I am concerned about the effectiveness of the COVID-19 vaccine					
	14. I worry that the vaccination will worsen my cancer condition					
	15. I think vaccination may interrupt the effectiveness of cancer treatment					
**Cues to action**	16. I'm willing to receive the COVID-19 vaccine if my doctor recommends it					
	17. I'm willing to receive the COVID-19 vaccine if the media recommends it					
	18. I'm willing to receive the COVID-19 vaccine if my family, friends and peers recommend it					

Table S2. Status of COVID-19 Vaccines within WHO EUL/PQ evaluation process (update on 07 July 2022)[1]

Manufacturer	Name of Vaccine	NRA of Record	Platfrom	EOI accepted	Pre-submission meeting held	Dossier accepted for review	Status of accessment	Decision date
Sinopharm / BIBP<br>Beijing Institute of Biological Products Co., Ltd. (BIBP)	SARS-CoV-2 Vaccine (Vero Cell), Inactivated (lnCoV)	NMPA	Inactivated, produced in Vero cells	√	√	√	Finalized 2 and 5 dose presentation (new manufacturing site)	07 May 2021<br>28 December 2021
sinovac<br>Sinovac Life Sciences Co., Ltd.	COVID-19 Vaccine (Vero Cell), Inactivated/ Coronavac™	NMPA	Inactivated, produced in Vero cells	√	√	√	Finalized 2 dose presentation	01 June 2021<br>30 September 202
康希诺生物 CanSinoBIO<br>CanSinoBIO	Ad5-nCoV	NMPA	Recombinant Novel Coronavirus Vaccine (Adenovirus Type 5 Vector)	√	√	Rolling data started 09 August 2021	Finalized	19 May 2022
Sinopharm / WIBP<br>Wuhan Institute of Biological Products Co., Ltd. (WIBP)	Inactivated SARS-CoV-2 Vaccine (Vero Cell)	NMPA	Inactivated, produced in Vero cells	√	√	Rolling data started 23 July 2021	Ongoing	To be confirmed
Zhifei Longcom, China	Recombinant Novel Coronavirus Vaccine （CHO Cell)	NMPA	Recombinant protein subunit	√	2 Pre-submission meeting held on 1 and 21 Dec 2021	Rolling data started 28 March 2022	Ongoing	To be confirmed

NRA: National Regulatory Authorities

MNPA: National Medical Products Administration

[1] Status of COVID-19 Vaccines within WHO EUL/PQ evaluation process. https://extranet.who.int/pqweb/sites/default/files/documents/Status_COVID_VAX_07July2022.pdf (update on **07 July 2022**).
